# Predicting patients with septic shock and sepsis through analyzing whole-blood expression of NK cell-related hub genes using an advanced machine learning framework

**DOI:** 10.3389/fimmu.2024.1493895

**Published:** 2024-11-28

**Authors:** Chao Du, Stephanie C. Tan, Heng-Fu Bu, Saravanan Subramanian, Hua Geng, Xiao Wang, Hehuang Xie, Xiaowei Wu, Tingfa Zhou, Ruijin Liu, Zhen Xu, Bing Liu, Xiao-Di Tan

**Affiliations:** ^1^ Department of Gastroenterology, Weihai Municipal Hospital of Shandong University, Weihai, Shandong, China; ^2^ Department of Pediatrics, Feinberg School of Medicine, Northwestern University, Chicago, IL, United States; ^3^ Department of Gastroenterology, Linyi People’s Hospital, Weifang Medical University, Linyi, Shandong, China; ^4^ Loyola University Chicago Stritch School of Medicine, Maywood, IL, United States; ^5^ Center for Pediatric Translational Research and Education, Department of Pediatrics, College of Medicine, University of Illinois at Chicago, Chicago, IL, United States; ^6^ Department of Biomedical Sciences and Pathobiology, Virginia-Maryland College of Veterinary Medicine, Blacksburg, VA, United States; ^7^ Department of Statistics, Virginia Tech, Blacksburg, VA, United States; ^8^ Department of Critical Care Medicine, Linyi People’s Hospital, Weifang Medical University, Linyi, Shandong, China; ^9^ Department of Research & Development, Jesse Brown Veterans Affairs Medical Center, Chicago, IL, United States

**Keywords:** sepsis, septic shock, biomarkers, machine learning for disease diagnosis, translational medicine, SepxFindeR model

## Abstract

**Background:**

Sepsis is a life-threatening condition that causes millions of deaths globally each year. The need for biomarkers to predict the progression of sepsis to septic shock remains critical, with rapid, reliable methods still lacking. Transcriptomics data has recently emerged as a valuable resource for disease phenotyping and endotyping, making it a promising tool for predicting disease stages. Therefore, we aimed to establish an advanced machine learning framework to predict sepsis and septic shock using transcriptomics datasets with rapid turnaround methods.

**Methods:**

We retrieved four NCBI GEO transcriptomics datasets previously generated from peripheral blood samples of healthy individuals and patients with sepsis and septic shock. The datasets were processed for bioinformatic analysis and supplemented with a series of bench experiments, leading to the identification of a hub gene panel relevant to sepsis and septic shock. The hub gene panel was used to establish a novel prediction model to distinguish sepsis from septic shock through a multistage machine learning pipeline, incorporating linear discriminant analysis, risk score analysis, and ensemble method combined with Least Absolute Shrinkage and Selection Operator analysis. Finally, we validated the prediction model with the hub gene dataset generated by RT-qPCR using peripheral blood samples from newly recruited patients.

**Results:**

Our analysis led to identify six hub genes (*GZMB, PRF1, KLRD1, SH2D1A, LCK*, and *CD247*) which are related to NK cell cytotoxicity and septic shock, collectively termed 6-HubG_ss_. Using this panel, we created SepxFindeR, a machine learning model that demonstrated high accuracy in predicting sepsis and septic shock and distinguishing septic shock from sepsis in a cross-database context. Remarkably, the SepxFindeR model proved compatible with RT-qPCR datasets based on the 6-HubG_ss_ panel, facilitating the identification of newly recruited patients with sepsis and septic shock.

**Conclusions:**

Our bioinformatic approach led to the discovery of the 6-HubGss biomarker panel and the development of the SepxFindeR machine learning model, enabling accurate prediction of septic shock and distinction from sepsis with rapid processing capabilities.

## Introduction

Sepsis remains the primary cause of in-hospital fatalities globally (1). The COVID-19 pandemic has underscored the urgency for diagnosis and treatment of sepsis. Timely and accurate identification of patients with sepsis is paramount for initiating early interventions, aligning with international consensus to enhance patient outcomes and lower mortality rates (2). Septic shock is the most severe manifestation of sepsis. Foreseeing this clinical condition has long been a focal point. Clinical studies show that each hour of delayed treatment in septic shock escalates the risk of death by approximately 8% (3). Consequently, discovering novel biomarkers and establishing effective predictive models for early septic shock detection is imperative, extending the window for prompt intervention.

Transcriptomics data have become advanced resources for identifying associations between gene expression levels and disease phenotypes and endotypes (4–8). However, due to its high-dimensional and complex features, analyzing such data can be challenging. The NCBI Gene Expression Omnibus (GEO) is an excellent resource for retrieving gene expression data, including data related to disease diagnosis and prognosis (9). Through the analysis of large-scale GEO datasets, insights into differentially expressed genes and pathways associated with specific diseases can be gained, allowing for the development of biomarkers for diagnosis and treatment based on this information. Recently, an increasing number of studies have used numerous one-step machine learning approaches to leverage existing large-scale gene expression datasets to establish biomarker prediction models for disease diagnosis and endotyping (10, 11). However, these models are currently awaiting validation through essential strategies to assess their accuracy and robustness across diverse datasets and patient demographics.

**Figure 1:**
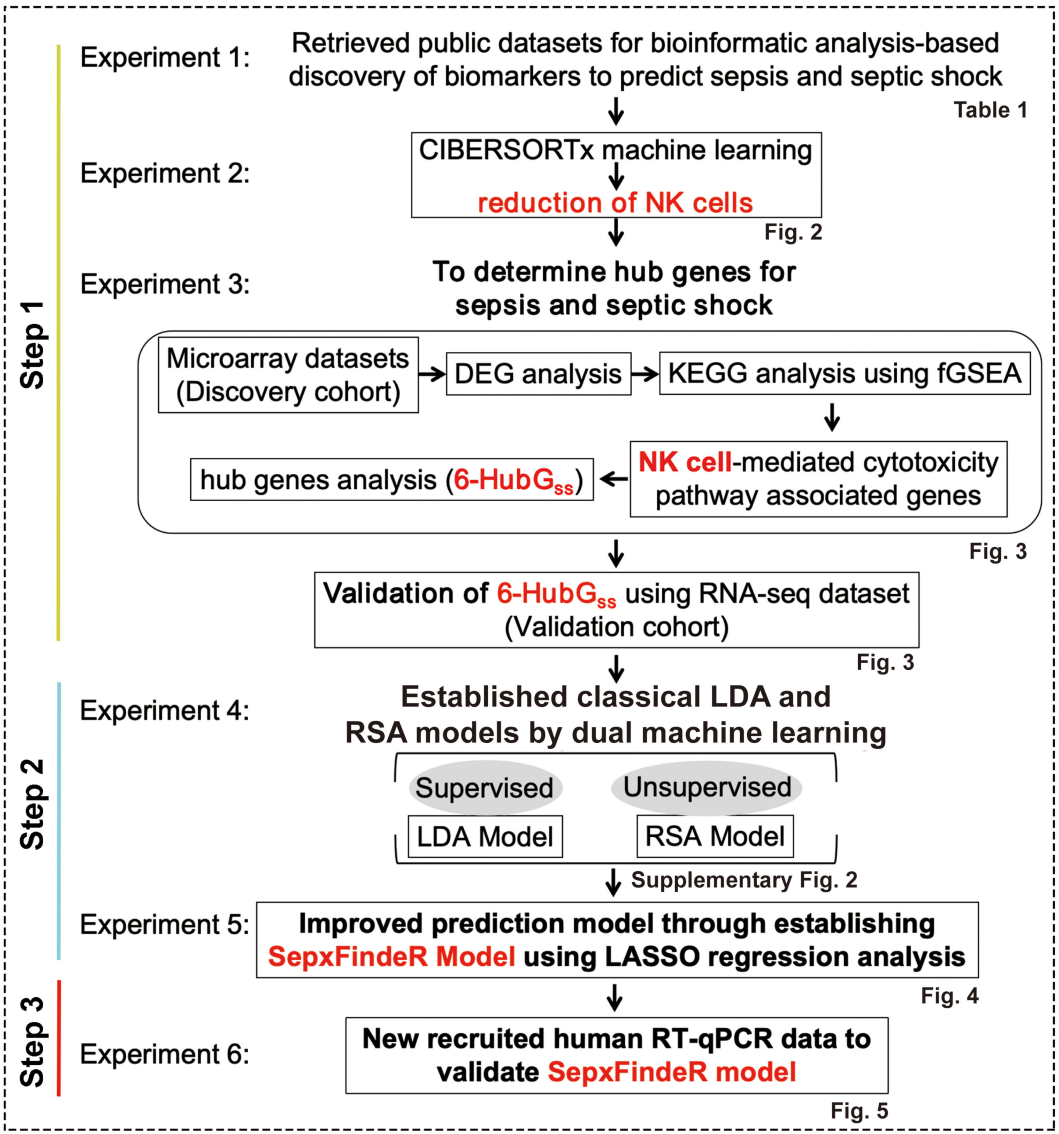
Workflow of model generation and validation. LDA, linear discriminant analysis. RSA, risk score analysis.

In this study, our objective is to establish a novel machine learning framework, called SepxFindeR (i.e. finding of patients with sepsis and septic shock) for prediction of sepsis and septic shock with rapid turnaround methods (RT-qPCR). To accomplish this goal, we executed a multistep workflow including (i) to develop an advanced approach for discovering a biomarker panel for septic shock using public transcriptome datasets, (ii) to establish the SepxFindeR model using a multistage machine learning algorithm to distinguish sepsis from septic shock with the identified biomarker panel, and (iii) to validate the SepxFindeR model using a dataset derived from the RT-qPCR test (**Figure 1**). This advanced workflow holds the potential to revolutionize the field of medicine by facilitating rapid disease diagnosis, paving the way for personalized treatment plans, and enhancing patient outcomes.

## Methods

### Study design

The NCBI GEO is a publicly available database containing vast amounts of human gene expression metadata that can be re-analyzed for translational research in advancing the prevention, diagnosis, or treatment of diseases (12). Our goal was to identify a biomarker panel related to sepsis and septic shock through analysis of the metadata in GEO. We then used a bioinformatics and machine learning approach to establish a highly predictive model to distinguish between septic shock, sepsis, and healthy individuals. To achieve this, we executed a pipeline consisting of six experiments outlined in **Figure 1**.

### Search and retrieval of gene expression metadata

We searched GEO of human datasets related to adult and pediatric populations. For adult datasets, a manual search of GEO repository (http://www.ncbi.nlm.nih.gov/geo/) was conducted with the following string: (((“shock, septic”[(MeSH Terms]) OR septic shock[(All Fields])) OR (“sepsis”[(MeSH Terms]) OR sepsis[(All Fields]))) AND (whole[(All Fields]) AND (“blood”[(Subheading]) OR “blood”[(MeSH Terms]) OR blood[(All Fields])))) AND “Homo sapiens”[(porgn])) AND “gse”[(Filter]) AND “Expression profiling by array”[(Filter])) AND “gse”[(Filter]). Next, all identified metadata in GEO repository were further assessed to determine if they consisted of (a) studies involved the use of adult whole blood specimens, (b) studies contained septic shock or sepsis patients with healthy controls, and (c) studies had blood samples collected within 24h of admission. Using these criteria, three transcriptional microarray datasets [GSE95233 (13), GSE57065 (14) and GSE54514 (15)] and one RNAseq dataset [GSE154918 (16)] were retrieved from the GEO. The features of these microarray and bulk RNAseq datasets are summarized in **Table 1**.

**Table 1:** Demographics of retrieved datasets.

GEO Accession ID	GSE95233		GSE57065		GSE54514		GSE154918		
Cohorts	Septic Shock ^**^	Healthy Control	Septic Shock ^**^	Healthy Control	Sepsis ^**^	Healthy Control	Septic Shock ^***^	Sepsis ^***^	Healthy Control
Time of sample collection* (n)	Day 1 (51)	Day 1 (22)	Day 1 (28)	Day 1 (25)	Day 1 (35)	Day 1 (18)	Day 1 (19)	Day 1 (20)	Day 1 (40)
Number of females	18	11	9	20	21	12	8	12	23
Number of males	33	11	19	5	14	6	11	8	17
Sample type	Whole blood cells		Whole blood cells		Whole blood cells		Whole blood cells		
Analysis platform	(HG-U133_Plus_2) Affymetrix Human Genome U133 Plus 2.0 Array		(HG-U133_Plus_2) Affymetrix Human Genome U133 Plus 2.0 Array		Illumina HumanHT-12 V3.0 expression beadchip		RNAseq		
Platform spot No.	GPL570		GPL570		GPL6947				
Comparison	Septic shock vs. Healthy Control		Septic shock vs. Healthy Control		Sepsis vs. Healthy Control		Septic shock vs. Sepsis, Septic shock vs. Healthy Control, Sepsis vs. Healthy Control		
References	(13)		(14)		(15)		(16)		

*The day or minutes after first onset of the disorder or visited clinics.

**Diagnosed using the diagnostic criteria of the American College of Chest Physicians/Society of Critical Care Medicine (1992).

***Diagnosed using the diagnostic criteria of The Third International Consensus Definitions for Sepsis and Septic Shock (Sepsis-3) (2016).

The gene expression level in microarray and RNAseq datasets is represented as fold change vs. RMA (Robust Multi-array Average) and TPM (Transcript per million) respectively, which are already normalized and can be comparable across samples within the same dataset in subsequent analysis.

### Evaluation of cell type abundance in retrieved gene expression datasets by CIBERSORTx algorithm

We used the CIBERSORTx algorithm, an established machine-learning RNA deconvolution method that infers cell-type-specific gene expression profiles (17), to estimate the proportions of leukocytes in each retrieved gene expression dataset. The retrieved transcriptome datasets were uploaded as mixture files to the CIBERSORTx web portal (https://cibersortx.stanford.edu/) (17), and the LM22 signature matrix was used to define the cell populations during the deconvolution analysis (17, 18). The algorithm was run with default parameters and 100 permutations in relative quantification. Deconvoluted samples were considered significant if the CIBERSORTx *p*-value was < 0.05, indicating a good fit across all cell subsets. The data output from CIBERSORTx was downloaded and analyzed using R programming language. Differences between lesion types were analyzed using independent Student’s t-test or one-way ANOVA followed by Tukey’s HSD *post-hoc* test. Results were presented as mean ± standard error of mean (s.e.m.), and a *p*-value < 0.05 was considered significant. Overall, the CIBERSORTx algorithm enabled us to estimate the relative proportions of leukocyte cell types in the blood samples of each individual in the retrieved dataset.

### Differentially expressed gene analysis and biological interpretation

The retrieved gene expression data were processed using the limma package in R software (Version 4.1.0 https://cran.r-project.org/web/packages/glmnet/index.html), and adjusted *p*-value<0.05 and |logFC| >0.6 were used to identify differentially expressed genes (DEGs) between defined conditions. To gain insights into the biological functions of the identified DEGs in the context of biological pathways and processes they are involved in, the septic shock-associated 1639 common DEGs in GSE95233 and GSE57065 were subjected to Kyoto Encyclopedia of Genes and Genomes (KEGG) pathway enrichment analysis using the Fast Gene Set Enrichment Analysis (fGSEA) package, which ranked genes based on the fold-change of their differential expression and visualized leading edge gene sets in the identified pathways. The identified top leading-edge genes were defined as hub-gene panel which was used for establishing predictive models through machine learning analysis.

### Establishment and assessment of hub gene panel using machine learning analysis

The following two machine learning analysis methods were executed to assess hub-gene panel in this study.

#### Linear discriminant analysis (LDA)

The identified hub-gene panel was used to build a prediction model by incorporating the LDA machine learning algorithm (10, 19). Briefly, the workflow for LDA analysis includes randomly splitting data into training set and test set with a ratio of 50/50, training the LDA model for discriminating sepsis and septic shock using the training set and evaluating predictive value of the model using the test set. The performance of the hub-gene guided model is evaluated using confusion matrix and receiver operating characteristic (ROC) curve.

#### Risk score analysis (RSA)

The risk score-related predictive model is a scoring system that represents a linear combination of the relative expression values of genes, with a weight value for unsupervised classification (10). To execute the analysis, the microarray datasets were processed using unsupervised machine learning, and a septic shock risk score was assigned to each individual based on the expression levels of the 6 HubG_ss_. The formula corresponding to the expression and risk score is as follows:


$$\text{Risk Score}=\sum_{i=1}^{n}w_{i}\left(\frac{e_{i}-u_{i}}{s_{i}}\right)$$

(n: count of 6-HubG_ss_; w: weight value of the i^th^ gene; e_i_: expression level of the i^th^ gene; u_i_: mean value for the i^th^ gene among whole samples; s_i_: standard deviation value for the i^th^ gene among whole samples.) The results of RSA were interpreted by evaluating the performance of the hub-gene guided risk score model. A density plot was used to determine the cutoff value, and an ROC curve was employed to estimate the specificity and sensitivity.

### The Least Absolute Shrinkage and Selection Operator analysis and establishment of SepxFindeR prediction model

The Least Absolute Shrinkage and Selection Operator (LASSO) regression analysis is one of the popular techniques used to improve machine learning model performance on small sample size and high-dimensional data (20). LASSO algorithm performs linear regression analysis on a complex dataset with multiple variables. It uses regularization to prevent overfitting by shrinking small coefficients of the predictor variables towards zero. In this study, the package “glmnet” in the R Programming Language (version 4.1) was used to carry out LASSO regression analysis to assess the relationship between disease categories and DEG expression levels of 6 hub genes. The workflow of LASSO analysis includes the following four steps. First, we established dataset, namely, Dataset^LD1+RS^, by treating the LD1 values from LDA analysis and risk scores from RSA analysis as two predictive variables while keeping disease categories as the response variable. The purpose is to borrow strength from both LDA and RSA by integrating the two informative features. This Dataset^LD1+RS^ was split into training and test sets. Second, we process the training dataset to train a LASSO regression model, namely, SepxFindeR using the glmnet package in R with parameters: family = “binomial”, type.measure = “deviance”, nfold = 20 (20-fold cross-validation). Specify the alpha parameter for L1/L2 regularization, with a=1 representing LASSO and a=0 representing ridge regression. Use the cv.glmnet function in R to perform cross-validation and select the optimal value for turning parameter l using “deviance”(-2 log partial likelihood). The λ was chosen so that the partial likelihood deviance reached its the lowest level. A suitable model was chosen based on the 20-fold cross-validation of the function cv.glmnet. Third, we evaluated the performance of the SepxFindeR model using the test dataset in terms of classification of accuracy. Lastly, we used the SepxFindeR model to make predictions on new datasets and compared the performance of the SepxFindeR model to that of the LDA and RSA models.

### Animal experiments and cecal ligation and puncture (CLP)-induced sepsis in mice

The protocol for animal experiments was approved by the Institutional Animal Care and Use Committee at Northwestern University. Specific pathogen free C57BL/6 wild-type mice (male, 8 weeks old) were purchased from Jackson Laboratory (Bar Harbor, ME). All mice were housed under a 12-h light-dark cycle with unlimited water and standard rodent chow in a specific pathogen-free environment. Mice were subjected to model of CLP-induced sepsis using our standard protocol (21, 22). Sham-control group received sham operation. The total number of mice used was 34. We used at least 7 mice in each group based on Power analysis. Mice were randomized into each experimental group and processed for treatments using a memory-free and pseudo-random selection process. They were monitored to determine body weights and the disease activity index (DAI) daily. The criteria of DAI for sepsis are detailed in **Supplementary Table S1**. The score of body weight was scaled as follows: weight loss (%): 0, normal; 1, <10%; 2, 10-15%; 3, 15-20%; 4, >20%, while the sepsis score was scaled based on murine sepsis score (MSS) as described previously (23): 0, MSS=0; 1, MSS<7; 2, MSS≥7 but <14; 3, MSS≥14 but <21; 4, MSS≥21. Then DAI was calculated as the sum of body weight score and sepsis score. At the end of experiments, mice were euthanized using CO_2_ inhalation followed by bilateral pneumothorax or cervical dislocation, and the blood samples were collected. No mice, samples, or data points were excluded from analyses. All evaluators were blinded to mouse treatment groups.

### Flow cytometry

We used flow cytometry to characterize the immune cells in mouse peripheral blood using our previously established protocol (24). The antibodies listed in **Supplementary Table S2** was used for immunostaining. The data were obtained using a BD FACSymphony A5 Cell Analyzer (Indianapolis, IN). Cells were first gated for FSC-A vs. SSC-A based on size and granularity to eliminate debris and clumped cells. Next, single cells were obtained using FSC-A vs. FSC-H and SSC-A vs. SSC-H gating strategy. These single cells were further sub-gated using the fixable live-dead viability dye for gaining live cells. Live cells were further gated for leukocyte cells based on the pan-hematopoietic marker CD45. Live CD45^+^ cells were used for the characterization of further immune cell subtypes. CD45^+^ cells were subjected to CD11b gating. The CD11b^+^ and CD11b^-^ cell populations were further gated to separate neutrophils (CD45^+^CD3^-^CD11b^+^Ly6g^+^), monocytes (CD45^+^CD3^-^CD11b^+^Ly6c^+^), NK cells (CD45^+^CD3^-^CD11b^+^NK1.1^+^) and T cells (CD45^+^CD11b^-^CD3^+^). Antibodies were titrated by performing fluorescence minus one (FMO). The flow cytometry data was then analyzed using FlowJo version 10.7.1 (FlowJo LLC, Ashland, OR).

### Human subjects and samples collection

In our study, we recruited a total of 18 healthy individuals and 28 patients diagnosed with septic shock (n=13) and sepsis (n=15). Ethical and research governance approval was provided by the Human Research Ethics Committee in Linyi People’s Hospital (Linyi, Shandong Province, China). Full study protocol can be accessed upon request. Patients with sepsis or septic shock were enrolled from intensive care unit (ICU) in Linyi People’s Hospital from November 1, 2021 to November 31, 2022. Patients were eligible if they were enrolled to ICU within 24 h and aged 18 years older and less than 85 years old. None of recruited patients were found to be suffered from COVID19 in this study. The subjects were assigned to sepsis group based on their Sequential Organ Failure Assessment (SOFA) score on admission (≥2) in accordance with the Third International Consensus Definitions for Sepsis and Septic Shock (Sepsis-3) (2). Recruited patients with septic shock had to fulfill the above sepsis criteria with a vasopressor requirement to maintain the blood pressure and having a serum lactate level > 2 mmol/L in the absence of hypovolemia. Healthy controls (n = 18) were enrolled at Linyi People’s Hospital. Exclusion criteria: (1) subjects below the age of 18 years old and over 85 years old; (2) subjects with malignant tumors; (3) subjects with primary immunodeficiency, HIV and subjects under immunosuppressive drugs; and (4) inability to consent the subjects. The whole blood was collected to an anticoagulated tube on admission within 24 hours and stored at -80°C. Patient information is summarized in **Table 2**.

**Table 2:** Clinical characteristics.

	Healthy control	Sepsis^1^	Septic shock^1^	P value^3^ (vs. Ctr)
Age^2^ (Years)	60.85 ± 2.3	68.41 ± 4.26	66.77 ± 3.07	–
Gender (F/M)	7/12	5/10	8/5	
APACHE II Score	–	17.13 ± 5.5	21.92 ± 6.02	*P*<0.05
SOFA score	–	5.07 ± 2.08	6.15 ± 2.44	–
Glasgow score	–	13.73 ± 2.72	13.46 ± 1.6	–
Creatinine umol/L	–	144.33 ± 124.99	152.08 ± 122.79	–
Lactate mmol/L	–	1.34 ± 0.74	3.67 ± 1.49	*P*<0.001

^1^Diagnosed using the diagnostic criteria of The Third International Consensus Definitions for Sepsis and Septic Shock (Sepsis-3) (2016).^2^mean ± s.e.m.

^3^Student's t test or one-way ANOVA followed by Tukey’s HSD post-hoc test.

### Real-time quantitative PCR (RT-qPCR)

Total RNA was extracted from the blood samples using TRIzol reagent (Invitrogen) according to the manufacturer’s instructions. Reverse transcription was performed using the SuperScript™ First-Strand Synthesis (GeneCopoei). RT-qPCR was performed using BlazeTaq™ SYBR Green qPCR Mix 2.0 (GeneCopoei) according to the manufacturer’s manual. Primers were listed in **Supplementary Table S3**. The relative mRNA expression level of the 6-HubG_ss_ was calculated and normalized to the expression of glyceraldehyde-3-phosphate dehydrogenase (*GAPDH*) gene. The fold change of gene expression levels between samples was calculated using the 2^–ΔΔCT^ method.

### Validation of SepxFindeR model in RT-qPCR data

To validate the peripheral blood 6-HubG_ss_-based SepxFindeR prediction model, we first performed LDA model and risk score model based on the RT-qPCR data. LD1 values and risk scores were profiled to evaluate the predictive efficiency of the SepxFindeR model in separating septic shock or sepsis patients from healthy controls, as well as separating patients with septic shock from sepsis.

### Statistical analysis

All data are shown as mean ± s.e.m. Statistical analysis was performed with R software (version 4.1.0) or GraphPad Prism 8. The independent Student’s t-test, the nonparametric Mann–Whitney test, and one-way ANOVA followed by Tukey’s HSD post-hoc test were used to analyze the statistical significance of the group differences. The significance level was set at *p<0.05. **p < 0.01, ***p < 0.001, ****p < 0.0001.

No samples or data points were excluded from analyses.

## Results

### Overview of retrieved gene expression datasets

To conduct this study, an extensive search was carried out using specific keywords in the GEO database. The aim was to identify microarray and RNAseq gene expression data encompassing transcription profiles of whole peripheral blood cells in adult healthy individuals and patients diagnosed with sepsis (refer to the detailed Methods section). By July 2021, a total of 88 datasets were found in GEO, and upon careful evaluation, three microarray datasets and one RNAseq dataset were identified to meet the criteria outlined in the method section. These datasets were subsequently chosen for inclusion in this study. Notably, two datasets including GSE95233 (13) and GSE57065 (14) are cohorts with healthy individuals (HC) and patients with septic shock (SS), while GSE54514 dataset (15) contains healthy individuals (HC) and patients with sepsis (Sep). Additionally, GSE154918 provided an RNAseq dataset that includes individuals with SS, Sep, and HC (16). A comprehensive summary of the key information from these retrieved datasets is presented in **Table 1**.

### Reduction in peripheral blood NK cells is a notable feature for both humans and mice with septic disorder

**Figure 2:**
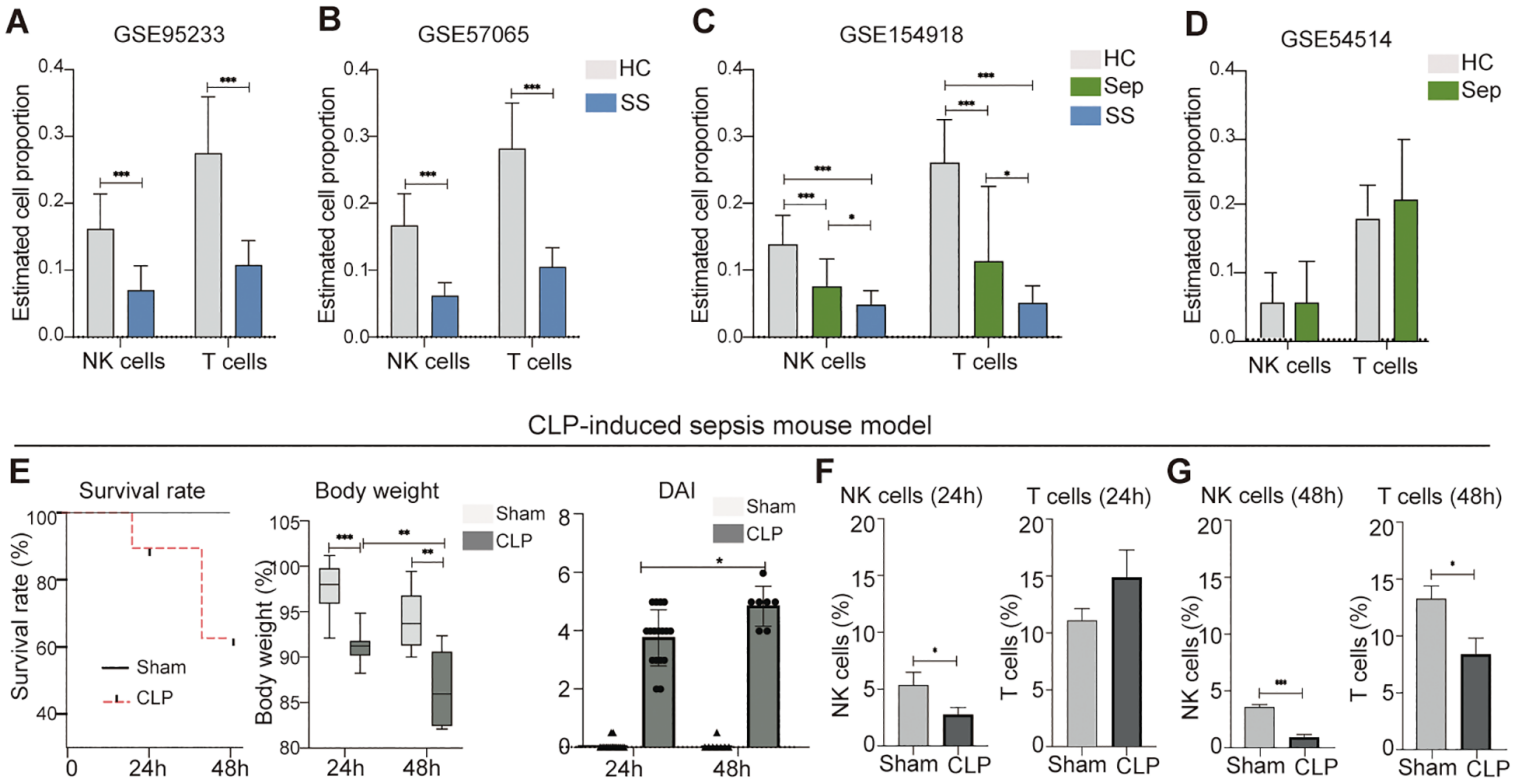
Reduction of peripheral blood NK cells is a distinctive feature of septic shock in mice and humans. **(A–D)** Comparison of NK cell and T cell proportions between individuals with sepsis and those who are healthy controls, septic shock vs. healthy controls, and septic shock vs. sepsis in datasets of GSE95233 **(A)**, GSE57065 **(B)**, GSE154918 **(C)**, and GSE54514 **(D)**. Relative proportion of NK cells and T cells in peripheral blood was estimated using CIBERSORTx machine learning platform. HC, healthy control; Sep, Sepsis; SS, septic shock. **(E)** Assessment of sepsis severity in indicated time periods after CLP based on survival rate, body weight, and disease activity (DIA). n = 17 in CLP 24 h group. n = 15 in Sham 24 h group. n = 7 in CLP 48 h group. n = 8 in Sham 48 h group. **(F, G)** Changes of NK cells and T cells in mouse peripheral blood at 24 h **(F)** and 48 h **(G)** after CLP. n = 7 in CLP 24 h group. n = 7 in Sham 24 h group. n = 7 in CLP 48 h group. n = 8 in Sham 48 h group. The proportions of indicated leukocytes in peripheral blood CD45^+^ cells were estimated by multicolor flow cytometry analysis. Data represents two independent experiments and show as mean ± s.e.m. *P < 0.05, **P < 0.01, ***P < 0.001. Student's t test or one-way ANOVA followed by Tukey’s HSD post-hoc test.

In order to utilize the retrieved datasets for this study, we thought to identify a specific peripheral blood cell population that is significantly affected by sepsis and septic shock. This would enable us to further investigate potential biomarkers associated with these conditions. To accomplish this, we employed the CIBERSORTx machine learning platform, a bioinformatic tool capable of retrospectively predicting the relative proportions of different cell types in a mixed cell population in peripheral blood using bulk RNA sequencing data (17). We applied this approach to estimate changes in the proportions of various types of peripheral leukocytes in human sepsis and septic shock datasets including GSE95233, GSE57065, GSE54514, and GSE154918. Our analysis revealed markedly reduction of NK cells and T cells in patients with septic shock (**Figures 2A–C**). In septic patients, RNAseq dataset GSE154918 showed a significant reduction in NK cells and T cells (**Figure 2C**), while microarray dataset GSE54514 displayed no significant changes in these two leukocyte subsets (**Figure 2D**). Thus, it appears patients with sepsis have database-dependent changes in NK cells and T cells. In addition, we found that the human sepsis and septic shock are associated with alteration of other leukocytes in a dataset dependent manner (**Supplementary Figure S1**). Similarly, we observed that male mice with CLP-induced septic disorder (**Figure 2E**) exhibited a persistent decrease in peripheral blood NK cells, but not other leukocytes during both the early sepsis stage (24 hours after CLP) and the severe sepsis stage (48 hours after CLP) (**Figures 2F, G**; **Supplementary Figure S1B**). As the goal of this study is not to characterize biomarkers for diagnosis of septic disorder for mice, we did not further confirm this finding using female mice. Collectively, our analysis suggests that both humans and mice exhibit a decrease in peripheral blood NK cells during the septic disorder.

### Patients with septic shock exhibit a significant decrease in the expression of hub genes related to NK cell cytotoxicity in peripheral blood cells

**Figure 3:**
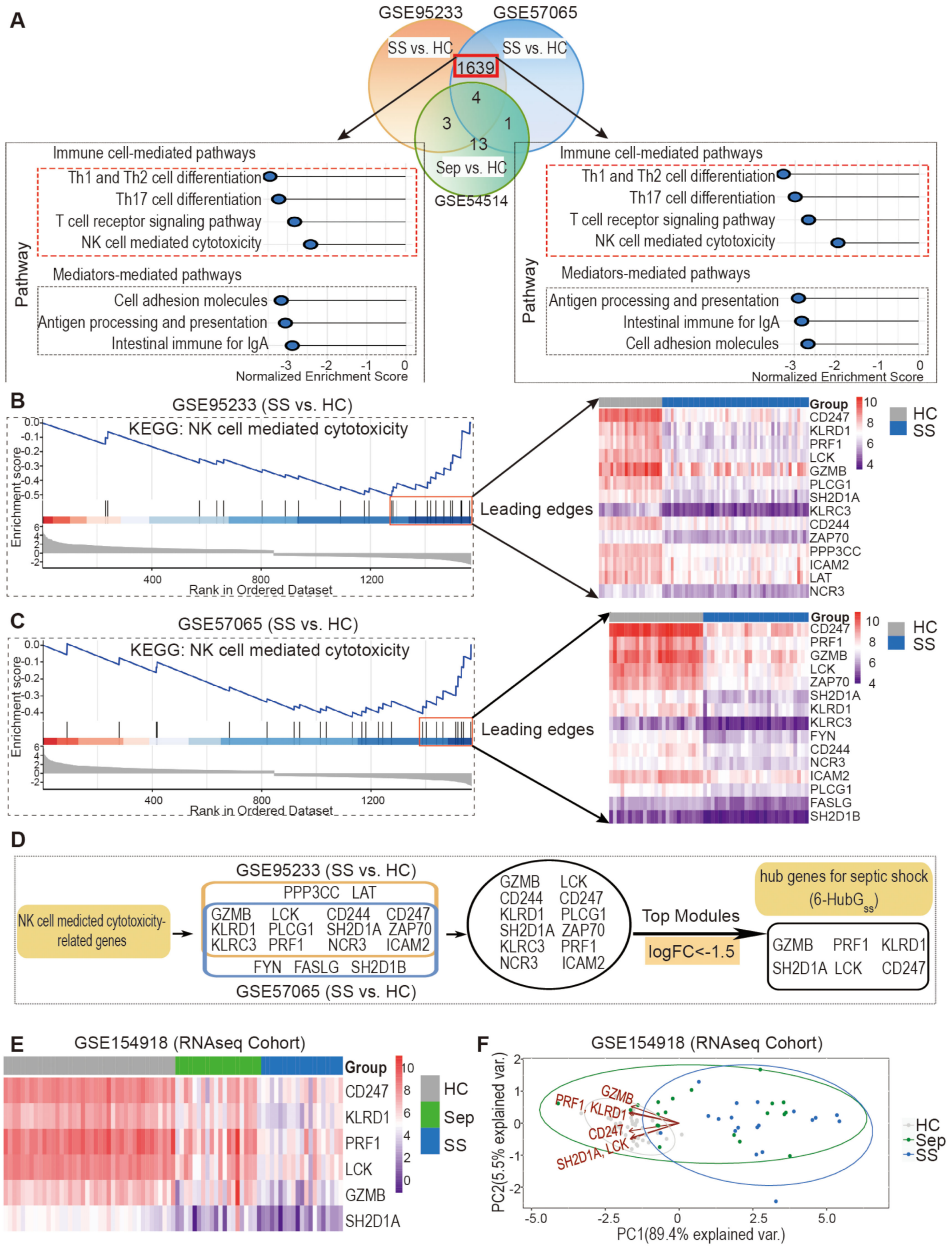
Discovery and validation of six hub genes for septic shock (6-HubG_ss_) through bioinformatic analysis of DEGs and KEGG related to NK cell mediated cytotoxicity. **(A)** DEG and KEGG analysis revealed septic shock-associated genes and pathways in datasets GSE95233 and GSE57065. GSE54514 dataset was used to exclude sepsis specific DEGs. **(B, C)** Identification of septic shock-associated hub genes through analysis of leading-edge genes in fGSEA profile of NK cell-mediated cytotoxicity pathway in GSE95233 **(B)** and GSE57065 **(C)**. **(D)** Unveiling a group of 6 top genes for septic shock (6-HubG_ss_) through top module analysis with leading edge genes in GSE95233 and GSE57065 datasets. **(E)** Validation of 6-HubG_ss_ in GSE154918 RNAseq dataset using heatmap analysis. **(F)** Assessment of performance of 6-HubG_ss_-guided PCA analysis on identification of patients with sepsis and septic shock in GSE154918 dataset. HC, healthy control; Sep, Sepsis; SS, septic shock.

Given the conserved changes observed in peripheral NK cell profiles in both mice and humans during the septic disorder, we hypothesized that NK cell-associated genes serve as candidate biomarkers for predicting sepsis-associated clinical conditions in humans. To test this hypothesis, we performed differential expression gene (DEG) and KEGG pathway analyses on retrieved microarray datasets, including GSE95233, GSE57065, and GSE54514, to investigate the association between changes in NK cell-related gene expression and sepsis/septic shock. Using R analytics, we analyzed each dataset to identify DEGs between septic shock patients and healthy controls, as well as between sepsis patients and healthy controls, using adjusted *P*<0.05 and |logFC| >0.6 as cutoff criteria. Notably, we found a higher number of DEGs between septic shock and healthy controls as compared to between sepsis and healthy controls (**Supplementary Table S1**). By employing Venn diagram analysis, we identified 1639 DEGs that were common in the GSE95233 and GSE57065 datasets when comparing septic shock patients to healthy controls, but these DEGs were not overrepresented in the GSE54514 dataset for sepsis patients (**Figure 3A**, top panel). Among these genes, 894 were upregulated and 745 were downregulated in both GSE95233 and GSE57065 (**Supplementary Table S2**).

To further explore the enriched pathways in septic shock patients compared to healthy controls, we conducted Gene Set Enrichment Analysis (GSEA) using the fGSEA R package and KEGG pathways in the GSE95233 and GSE57065 datasets. Our analysis revealed a significant enrichment of genes associated with cellular and humoral immune responses, as well as pre-existing health conditions, in septic shock patients (**Supplementary Table S3**). Notably, among the enriched cellular pathways, we observed a marked downregulation of signals associated with the T cell signaling pathway and NK cell cytotoxicity (**Figure 3A**, bottom panel). Based on these findings, we further processed bioinformatic analysis and identified potential hub genes for septic shock within the downregulated leading-edge genes from the NK cell-mediated cytotoxicity pathway (**Figures 3B, C**). By DEG analysis of the top leading-edge genes between the GSE95233 and GSE57065 datasets, we identified 12 overlapping candidate genes (**Figure 3D**). Utilizing a cutoff of LogFC<-1.5, we identified a group of six genes, which we designated as the six hub genes for septic shock (6-HubG_ss_) in peripheral blood cells (**Figure 3D**). Furthermore, we examined how the expression of the 6-HubG_ss_ is altered in samples from GSE154918 RNAseq dataset, an independent validation cohort. The heatmap shows that the six hub genes were significantly downregulated in septic shock patients as compared to heathy controls (**Figure 3E**). PCA analysis revealed that the 6-HubG_ss_ biomarker panel effectively segregates patients with sepsis and septic shock from the healthy control cohort (**Figure 3F**). However, the predictive efficiency of 6-HubG_ss_ panel-based PCA analysis is lacking in its ability to differentiate between septic shock and sepsis (**Figure 3F**). This suggests that 6-HubG_ss_ panel-based PCA analysis is not suitable to distinguish septic shock from sepsis.

### Evaluating the reliability of HubG_ss_ panel with LDA and RSA analyses

Here, we employed a dual machine learning approach in R (v.4.1.0) to evaluate the potential of the 6-HubG_ss_ panel as a reliable biomarker for predicting septic shock and sepsis using a workflow illustrated in **Supplementary Figure S2A**. First, the retrieved microarray datasets (GSE95233, GSE57065, and GSE54514) were assigned as the discovery-cohort. Utilizing 6-HubG_ss_ panel-guided LDA and RSA analyses, we constructed two biomarker models, namely the LDA^6-HubGss^ and RSA^6-HubGss^ models for analysis the discovery-cohort. Evaluation of performance metrics under a train-test split setting demonstrated that the LDA^6-HubGss^ model exhibited excellent predictive values in identifying patients with septic shock in the training and test groups in the GSE95233 and GSE57065 datasets (**Supplementary Figure S2B**, performance metrics in top panel). The measurement of the area under the ROC curve of LD1 value further revealed a perfect ROC score for the specificity/sensitivity pair of the LDA^6-HubGss^ model (**Supplementary Figure S2B**, ROC curves in top panel). However, the LDA^6-HubGss^ model showed insufficient accuracy in determining patients with sepsis in both training and test sets of the GSE54514 dataset (**Supplementary Figure S2C**, performance metrics in top left panel), with a poor/failed ROC score (**Supplementary Figure S2C**, top right panel). Similarly, the RSA^6-HubGss^ model enables to separate patients with septic shock from healthy control in each dataset based on the expression levels of the 6-HubG_ss_ (**Supplementary Figure S2B**, density plot in bottom panel). The specificity and sensitivity of the RSA^6-HubGss^ model were verified using ROC curve analysis, demonstrating unbiased prediction of septic shock patients in the microarray datasets using the 6-HubG_ss_-guided machine learning bioinformatic approach (**Supplementary Figure S2B**, ROC curves in bottom panel). However, the RSA^6-HubGss^ model was observed to be incapable of distinguishing between sepsis patients and healthy individuals in the GSE54514 dataset (**Supplementary Figure S2C**, bottom panel).

Next, we conducted a series of cross-validation analyses to verify the accuracy of LDA^6-HubGss^ and RSA^6-HubGss^ models in analyzing an independent validation cohort of the GSE154918 RNAseq dataset, which consisted of individuals with septic shock, sepsis, and healthy controls. In the LDA-based cross-validation analysis, we observed excellent prediction accuracy of the LDA^6-HubGss^ model for distinguishing septic shock from healthy controls (**Supplementary Figure S2D**, performance metrics in top panel) and sepsis from healthy controls (**Supplementary Figure S2E**, performance metrics in top panel) in both the training and test groups of the GSE154918 dataset. However, when assessing the confusion matrix for septic shock versus sepsis, we found that the LDA^6-HubGss^ model excelled on the training data but did not generalize effectively to the test data (**Supplementary Figure S2F**, performance metrics in top panel). Additionally, we repeated the evaluation analysis using all subjects in both the training and test sets. The ROC curves demonstrated that the LD1 value derived from the LDA^6-HubGss^ model showed excellent discriminatory performance for sepsis and septic shock compared to healthy controls, with AUC (Area Under the Curve) values of 0.959 and 1, respectively (**Supplementary Figures S2D**, **S2E**, ROC curves in top panel). In contrast, the AUC value for sepsis versus septic shock was 0.832 (**Supplementary Figure S2F**, ROC curves in top panel), suggesting that the LDA^6-HubGss^ model is ineffective in discriminating between sepsis and septic shock.

Furthermore, the GSE154918 dataset was applied on RSA^6-HubGss^ model to distinguish sepsis and septic shock. Risk score distribution plot shows separation of patients with septic shock (**Supplementary Figure S2D**, histogram in bottom panel) and sepsis (**Supplementary Figure S2E**, histogram in bottom panel) from healthy individuals in the GSE154918 dataset by RSA^6-HubGss^ model. Similarly, ROC curve analysis of the risk scores demonstrated that the RSA^6-HubGss^ model exhibited excellent predictive accuracy in identifying patients with septic shock (**Supplementary Figure S2D**, ROC curves in bottom panel) as well as sepsis (**Supplementary Figure S2E**, ROC curves in bottom panel) from healthy controls in the GSE154918 RNAseq dataset. However, both histogram plot of risk score distribution and ROC curve show that the RSA^6-HubGss^ model was unable to differentiate patients with septic shock from those with sepsis in the dataset (**Supplementary Figure S2F**, bottom panel). Together, it appears that 6-HubG_ss_ biomarker panel-based models of LDA and RSA exhibit limitations on segregating patients with septic shock from sepsis in gene expression omics data.

### Advancing models of LDA^6-HubGss^ and RSA^6-HubGss^ to SepxFindeR model using ensemble method combined with Least Absolute Shrinkage and Selection Operator regression analysis

**Figure 4:**
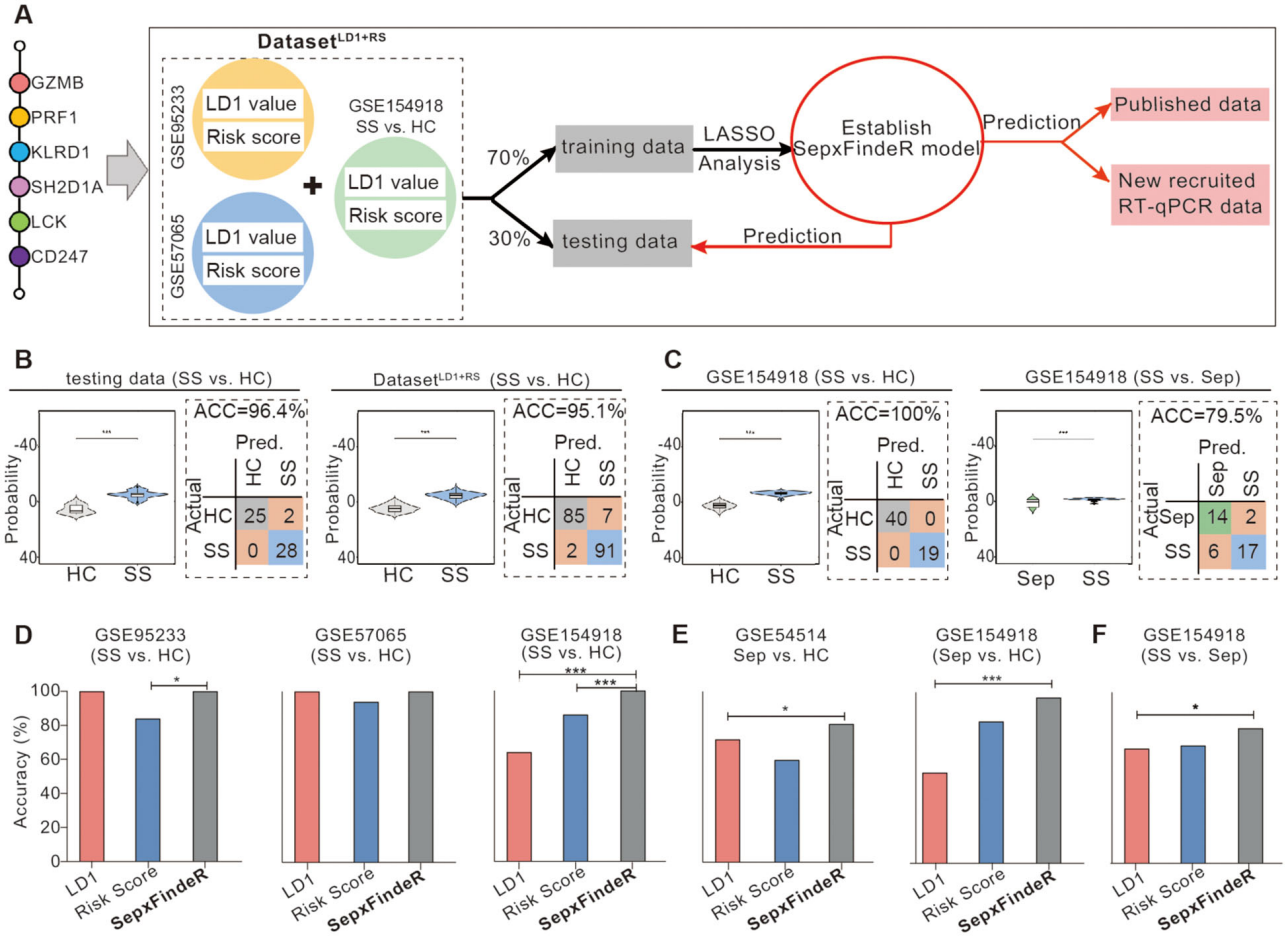
Establishment of SepxFindeR model using Least Absolute Shrinkage and Selection Operator (LASSO) analysis to improve the performance of 6-HubG_ss_ panel-guided prediction on septic shock and sepsis. **(A)** Workflow for building and verifying SepxFindeR model. **(B)** Assessment of SepxFindeR model in distinguishing patients with septic shock from healthy control in the testing set (left panel), merged data (right panel). **(C)** Assessment of SepxFindeR model in distinguishing patients with septic shock from healthy control (left panel) and sepsis (right panel) in GSE154918 dataset. **(D–F)** SepxFindeR model vs. LDA and RSA models. Comparison of the overall performance of SepxFindeR model with that of thresholds of LD1 values or cutoffs of risk scores from each dataset on distinguishing sepsis from healthy control **(D)**, septic shock from healthy control **(E)**, and septic shock from sepsis **(F)** in indicated datasets. HC, healthy control; Sep, Sepsis; SS, septic shock. *P < 0.05, ***P < 0.001. Student’s t-test or one-way ANOVA followed by Tukey’s HSD post-hoc test.

Ensemble method is a machine learning technique that combines several base models in order to produce one optimal predictive model (25). Here, we sought to examine whether this approach enables to improve LDA^6-HubGss^ and RSA^6-HubGss^ frameworks, leading to an advanced 6-HubG_ss_ panel-guided machine learning model which exhibits robust and reliable performance not only within individual datasets but also across multiple datasets. Thus, we employed an ensemble approach and performed LASSO regression analysis-based machine learning algorithm to improve LDA^6-HubGss^ and RSA^6-HubGss^ frameworks for predicting patients with sepsis and septic shock. To achieve this, we constructed a two-dimensional dataset called the 6-HubG_ss_ panel-associated Dataset^LD1+RS^ by merging the 6-HubG_ss_ panel-based LD1 values and risk scores for all individuals in the microarray datasets of GSE95233 and GSE57065 as well as for individuals of healthy controls and patients with septic shock in RNAseq dataset of GSE154918 (**Figure 4A**). Through the train-test split machine learning approach using LASSO regression analysis on the Dataset^LD1+RS^ dataset, we established a new machine learning prediction model, namely, SepxFindeR for predicting septic shock (**Figure 4A**). We found that the SepxFindeR model effectively distinguished patients with septic shock from healthy individuals in the test set and the full 6-HubG_ss_ panel-associated Dataset^LD1+RS^ (**Figure 4B**, violin plot for probability distribution). Confusion matrix analysis shows that SepxFindeR model exhibits an excellent performance in prediction of patients with septic shock in both test and merged dataset respectively (**Figure 4B**). Furthermore, we evaluated the SepxFindeR model using GSE154918 dataset that contains sepsis, septic shock, and healthy controls. We noticed SepxFindeR model exhibited an excellent performance on prediction of patients with septic shock vs. healthy controls (**Figure 4C**, left panel) and significantly separated patients with septic shock from sepsis in GSE154918 dataset (**Figure 4C**, right panel). The confusion matrix evaluation further demonstrated that the SepxFindeR model achieved an accuracy of 100% in predicting septic shock and 80% in differentiating septic shock from sepsis in GSE154918 dataset (**Figure 4C**).

Analysis of performance metrics demonstrated that predictive accuracy of SepxFindeR model is similar to that of the LDA and risk score models in terms of predicting septic shock in microarray datasets of GSE95233 (**Figure 4D**, left panel) and GSE57065 (**Figure 4D**, middle panel). In the GSE154918 RNAseq dataset, the SepxFindeR model exhibited much better performance in distinguishing septic shock compared to the LDA and RSA models in all analyzed datasets (**Figure 4D**, right panel). Compared to the LDA and RSA models, the SepxFindeR model demonstrated significantly better accuracy in predicting patients with sepsis in both microarray dataset (**Figure 4E**, left panel) and RNAseq dataset (**Figure 4E**, right panel). Among the three models, the SepxFindeR model exhibited the highest performance accuracy value for segregating septic shock from sepsis in the GSE154918 RNAseq dataset (**Figure 4F**). Together, these results suggest that the SepxFindeR model is an advanced machine learning model for predicting patients with sepsis and septic shock.

### SepxFindeR model effectively not only predicts sepsis and septic shock but also distinguishes them in 6-HubG_ss_ biomarker panel-based RT-qPCR dataset

**Figure 5:**
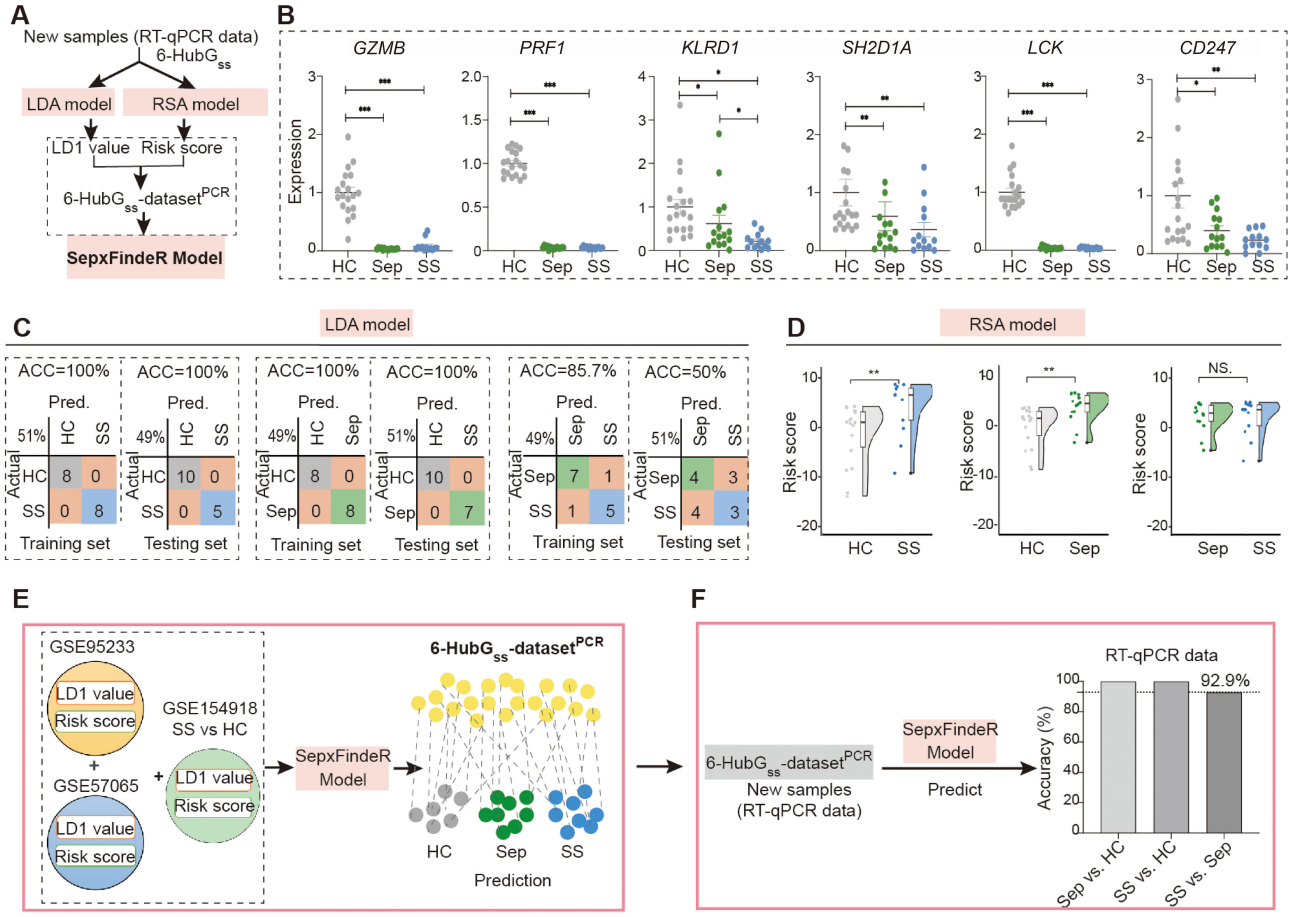
Application of LDA, RSA, and SepxFindeR models on prediction of patients with sepsis and septic shock in new 6 HubG_ss_ panel-based RT-qPCR dataset. **(A)** The workflow for analyzing RT-qPCR data using LDA, RSA, and SepxFindeR models. **(B)** Assessment of expression levels of 6 HubG_ss_ markers in peripheral blood cells of indicated individuals using RT-qPCR. n = 13-19/group. Data show as mean ± s.e.m. *P < 0.05, **P < 0.01, ***P < 0.001. (Sep vs. HC, SS vs. HC, SS vs. Sep: nonparametric Mann–Whitney test). **(C, D)** Distinguishing patients with sepsis and septic shock in 6 HubG_ss_ panel-based RT-qPCR dataset using LDA model **(C)** and RSA model **(D)**. **P<0.01. NS, no significance. **(E)** Overview about how to predict sepsis and septic shock in 6 the 6-HubG_ss_-dataset^PCR^ dataset using SepxFindeR model. **(F)** Application of SepxFindeR model to 6-HubG_ss_-dataset^PCR^ dataset. HC, healthy control; Sep, Sepsis; SS, septic shock.

In this study, we examined whether models of LDA, RSA, and SepxFindeR can be applied on prediction of patients with sepsis and septic shock in 6-HubG_ss_ biomarker panel-based RT-qPCR dataset through executing a workflow outlined in **Figure 5A**. For this purpose, we prospectively recruited 15 patients with sepsis and 13 patients with septic shock. Additionally, we enrolled 18 healthy individuals as a control cohort for this study. Upon enrollment, we collected peripheral blood cells for the purpose of this validation study. The patients were conventionally managed using a step-up strategy by clinicians who were blinded to the results of this validation study (**Table 2**). The peripheral blood cells were processed to extract total RNA, followed by measuring the expression of the 6-HubG_ss_ using RT-qPCR. Initially, we compared the expression of the 6-HubG_ss_ in patients with sepsis and septic shock to that in the healthy controls. In both septic conditions, the mRNA levels of the 6-HubG_ss_ were significantly lower than in the healthy controls (**Figure 5B**). However, no significant difference in the expression of the 6-HubG_ss_ was observed between patients with sepsis and septic shock. This suggests that the qPCR-based analysis of the 6-HubG_ss_ expression is unable to differentiate septic shock from sepsis.

Next, we processed the RT-qPCR-measured levels of the 6-HubG_ss_ in the cohort to construct a dataset called the 6-HubG_ss_-dataset^PCR^. We then executed LDA and RSA machine learning analyses using a train-test split routine to discriminate between sepsis and healthy individuals, septic shock and healthy individuals, and sepsis and septic shock in the 6-HubG_ss_-dataset^PCR^. The confusion matrices and kernel density plot reveal that both models effectively differentiated septic shock and sepsis from healthy individuals in the 6-HubG_ss_-dataset^PCR^ (**Figures 5C, D**, left and middle panels). However, neither the LDA model nor the RSA model can distinguish septic shock patients from those with sepsis (**Figures 5C, D**, right panel). This indicates that LDA model and the RSA model have limitations in differentiating septic shock from sepsis in 6-HubG_ss_-dataset^PCR^.

Finally, we processed the 6-HubG_ss_-dataset^PCR^ using SepxFindeR model to predict sepsis and septic shock (**Figure 5E**). Through confusion matrices analysis, we found that SepxFindeR model effectively identified all patients with sepsis and septic shock in the 6-HubG_ss_-dataset^PCR^ (**Figure 5F**) Remarkably, SepxFindeR machine learning segregated patients with septic shock from sepsis in 6-HubG_ss_-dataset^PCR^ with 92.9% accuracy (**Figure 5F**), suggesting SepxFindeR model possesses promise in predicting patients with sepsis and septic shock in comparison to healthy controls, as well as discriminating septic shock from sepsis in a dataset generated through a rapid turnaround RT-qPCR assay.

## Discussion

In this study, we developed SepxFindeR model, a novel machine learning framework tailored to distinguish between sepsis and septic shock patients by analyzing a specific set of NK cell-associated hub gene expressions in whole-blood samples. The methodology for creating SepxFindeR involves the meticulous execution of a comprehensive multi-step bioinformatic analysis (**Figure 1**). The SepxFindeR relies on the utilization of profiling these hub gene expressions in peripheral blood cells through RT-qPCR, a rapid quantitative method with a swift turnaround. This feature makes it an ideal candidate for RT-qPCR-based point-of-care test of whole blood samples, enhancing its clinical utility. Through the utilization of SepxFindeR, we have successfully showcased our ability to predict, with high accuracy, critically ill patients who face an elevated risk of progressing to sepsis and septic shock. Notably, the SepxFindeR machine learning framework not only enables precise identification of patients with sepsis and septic shock but also facilitates the differentiation between septic shock and sepsis cases. Together, the SepxFindeR machine learning framework holds the potential to significantly enhance the accuracy of differential diagnoses for sepsis and septic shock. Moreover, the procedural workflow used to establish SepxFindeR has the potential to be adapted for the creation of other machine learning frameworks designed to differentiate a range of diseases by analyzing transcriptome datasets.

Sepsis is a life-threatening syndrome of organ dysfunction induced by infection (2). It can be progressed to septic shock, a subgroup of sepsis wherein profound circulatory, cellular, and metabolic abnormalities are particularly pronounced. Septic shock leads to a higher mortality risk compared to sepsis alone. The Sequential Organ Failure Assessment (SOFA) scoring system has been widely employed to identify septic patients in clinical practice (26). Pinpoint of septic shock patients entails observing a need for vasopressors to maintain a mean arterial pressure of 65 mm Hg or higher, coupled with a serum lactate level exceeding 2 mmol/L (>18 mg/dL) in the absence of hypovolemia. However, the early diagnosis of sepsis and septic shock continues to present challenges. Therefore, it has become imperative to explore additional biomarkers that can facilitate recognizing these clinical conditions. The rapid advancements in high-throughput sequencing technology have generated extensive datasets, offering a promising avenue for identifying biomarkers that could significantly enhance early-stage diagnostics, prognostic assessments, and therapeutic strategies for a diverse range of medical conditions. Significantly, recent investigations have shed light on hub genes as pivotal components in sepsis diagnosis through bioinformatic analysis of sepsis-associated datasets, drawing insights from various gene expression profiles obtained from whole blood samples (27–29). A noteworthy example is the work of Lai et al. (30), in which they unveiled a cluster of seven hub genes exhibiting a robust correlation with sepsis. Likewise, Gano-Gamez et al., through a bioinformatic analytical approach, identified an additional set of hub genes associated with sepsis, enabling more precise patient stratification (31). However, these studies have yet to establish the potential of these hub genes in effectively distinguishing between patients with septic shock and those with sepsis. Compared to those methods, we demonstrated that SepxFindeR model enables to effectively discriminate septic shock from sepsis in not only RNAseq dataset but also RT-qPCR dataset.

Lymphopenia is a common occurrence in sepsis, and this particular aspect of pathophysiology has been recognized as a valuable predictive marker for the diagnosis of sepsis (32–34). However, there is a notable scarcity of prediction models centered around lymphopenia for the timely differential diagnosis of septic shock from sepsis. NK cells represent a specific subset of lymphocytes found in peripheral blood. In the present study, we have identified a reduction in the count of NK cells in peripheral blood during instances of sepsis and septic shock. This phenomenon remains consistent both in human subjects and in mice, suggesting that downregulation of genes associated with NK cells may hold substantial promise as potential biomarkers for predicting the occurrence of sepsis and septic shock. Building on this premise, we gathered extensive cohorts of sequencing data relevant to sepsis and septic shock from the GEO database. Subsequently, these datasets underwent bioinformatic analysis to explore the expression of genes linked to NK cells, to uncover biomarkers associated with septic shock. Our investigation unveiled that septic shock is indeed associated with a decrease in a group of 6 NK cell-associated hub genes including *GZMB, PRF1, KLRD1, SH2D1A, LCK*, and *CD247* (6-HubG_ss_) in peripheral blood samples. Notably, our data underscore the immediate translational potential of these 6-HubG_ss_-guided bioinformatic machine learning in differentiating instances of septic shock from sepsis, with a specific relevance to peripheral blood samples. This highlights the prompt translational promise inherent in this discovery.

The pursuit of biomarker development through machine learning is a harmonious blend of artistic intuition and scientific rigor, as the notion of a universally applicable singular solution or approach remains unequivocally absent. LDA- and RSA-based machine learning techniques are commonly employed as linear classifiers, finding extensive utility in confirming disease-associated signature genes obtained from bioinformatic analyses of omics data. While these statistical methodologies facilitate the detection of various disease-related molecular signatures, our investigation has revealed a limitation: none of these strategies adequately construct a machine learning prediction model guided by 6-HubG_ss_ for effectively distinguishing between septic shock and sepsis across different databases. In contrast, using a comprehensive methodology that involves the application of LDA and RSA, as well as harnessing the power of ensemble methods in conjunction with the LASSO machine learning approach, we developed the SepxFindeR model, leading to differential diagnosis between septic shock and sepsis. Ensemble-based approaches have demonstrated their efficacy particularly when dealing with datasets containing both linear and non-linear data types. Previously, Thrampoulidis et al. revealed that LASSO with non-linear measurements is equivalent to one with linear measurements (35). Therefore, we hypothesize that the relationship between the alteration in 6-HubG_ss_ expression and septic shock manifests in a non-linear fashion. Furthermore, we speculate that combining ensemble methods with the LASSO machine learning approach presents a viable alternative for discovering novel biomarkers from non-linear datasets, especially when a multi-base model approach proves ineffective.

We are mindful of the limitations inherent in this study. While we incorporated three microarray datasets and one RNAseq dataset, the inclusion of additional studies, particularly RNAseq datasets, is warranted to enhance the diversity of studies and amplify the sample size. Although we validated the SepxFindeR model using RT-qPCR dataset, it is important to note that this validation was based on a single center-associated study. To establish the robustness of SepxFindeR model, its validation should be extended to encompass data from multiple centers. Moreover, we encountered challenges in predicting septic shock from sepsis within the dataset derived from pediatric patients using SepxFindeR. In addition, we only used male mice to induce sepsis via CLP which may limit the generalizability of the finding in our study. Further investigation is necessary to address these challenges and refine the predictive capabilities of the model in this specific context.

In summary, our study indicates the significance of the 6-HubG_ss_ biomarker panel in relation to sepsis and septic shock. Through the evaluation of the expression profile of the 6-HubG_ss_ panel in whole-blood genes, employing a combination of LDA and RSA alongside ensemble methods and the LASSO machine learning approach, we enable to effectively differentiate septic shock from sepsis. This effort has led to the creation of SepxFindeR, a novel machine learning tool that facilitates identification of patients with septic shock using an RT-qPCR rapid turnaround method. This advancement takes us a step closer to realizing the potential of integrating machine learning technology and precision medicine for the management of patients with critical illness.

## Data Availability

The original contributions presented in the study are included in the article/**Supplementary Material**. Further inquiries can be directed to the corresponding author.

## Supplementary material

Supplementary Data Sheet 1 Supplementary Figures and Tables.

Supplementary Data Sheet 2 The checklist for flow cytometry study.

Supplementary Table 1 Excel file for DEGs of the three datasets on Day1.

Supplementary Table 2 Excel file for common DEGs in septic shock but not sepsis.

Supplementary Table 3 Excel file for the fGSEA pathways in datasets of GSE95233 and GSE57065.
