# Supplementary material for: Predicting patients with septic shock and sepsis through analyzing whole-blood expression of NK cell-related hub genes using an advanced machine learning framework
Source: Front Immunol. 2024 Nov 28;15:1493895. doi: 10.3389/fimmu.2024.1493895 (PMC11634752; doi:10.3389/fimmu.2024.1493895)
Supplement: Supplementary Data Sheet 1 — Supplementary Figures and Tables. [file DataSheet1.pdf]

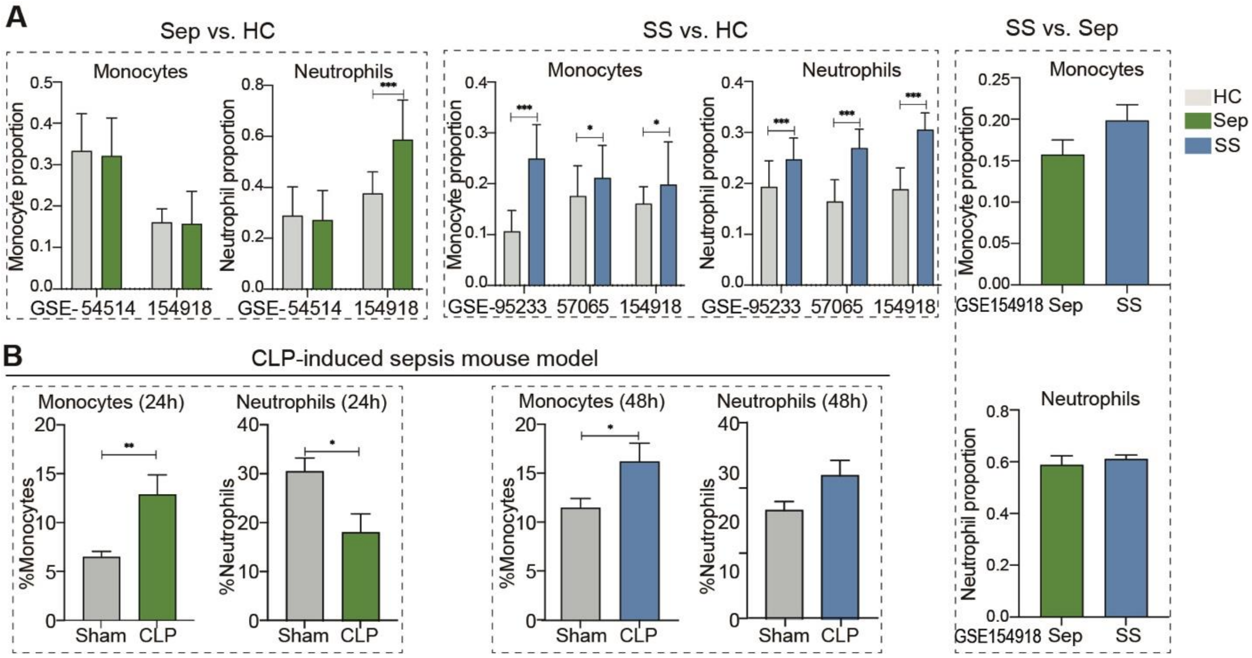

Supplementary Figure S1 Monocyte and neutrophil counts in peripheral blood of septic shock in mice and humans.

(A) Comparison of monocyte and neutrophil counts between individuals with sepsis and those who are healthy (left panel), septic shock vs. healthy (middle panel), and septic shock vs. sepsis (right panel) in indicated datasets. Relative proportion of NK cell and T cell in peripheral blood was estimated using CIBERSORTx machine learning platform. HC, healthy control; Sep, Sepsis; SS, septic shock. **(B)** Effect of CLP-induced sepsis (left panel) and septic shock (right panel) on monocyte and neutrophil counts in mouse peripheral blood. n = 7 in CLP 24 h group. n = 7 in Sham24 h group. n = 7 in CLP 48 h group. n = 8 in Sham 48 h group. Data represents two independent experiments and show as mean ± s.e.m. *$P < 0.05$, **$P < 0.01$, Student's t-test.

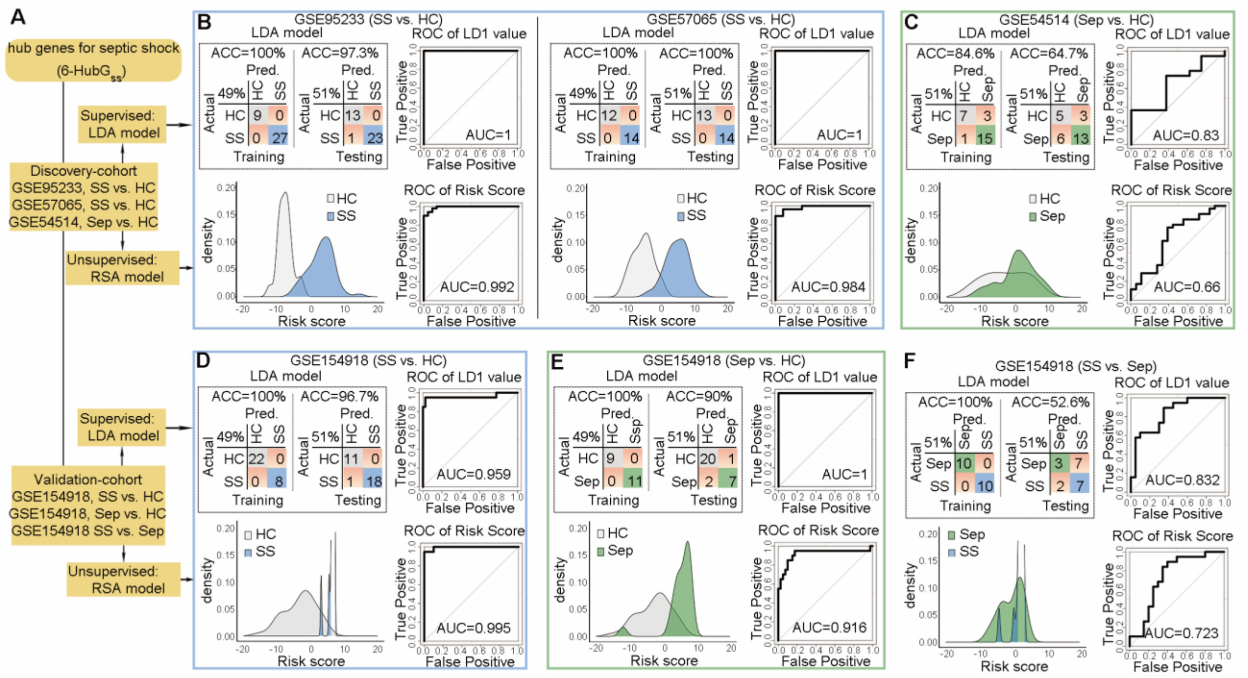

Supplementary Figure S2 Assessment of 6-HubG$_{ss}$ panel-based LDA and RSA machine learning models predicting sepsis and septic shock.

(A) Workflow for building LDA$^{6\text{-HubGss}}$ model and RSA$^{6\text{-HubGss}}$ dual machine learning models with 6-HubG$_{ss}$ panel for predicting sepsis and septic shock. **(B)** Assessment of 6-HubG$_{ss}$ panel-based LDA$^{6\text{-HubGss}}$ and RSA$^{6\text{-HubGss}}$ models in prediction of septic shock in discovery cohort of GSE95233 and GSE57065 datasets. **(C)** Assessment of 6-HubG$_{ss}$ panel-based LDA$^{6\text{-HubGss}}$ and RSA$^{6\text{-HubGss}}$ models in prediction of sepsis in discovery cohort of GSE54514 dataset. **(D - F)** Verifying 6-HubG$_{ss}$ panel-based LDA$^{6\text{-HubGss}}$ and RSA$^{6\text{-HubGss}}$ models in prediction of septic shock (D) and sepsis (E) as well as discriminating septic shock from sepsis (F) in validation cohort of GSE154918 dataset. HC, healthy control; Sep, Sepsis; SS, septic shock.

Supplementary Table S1. Sepsis-related disease activity index (DAI)

**BW* Loss**	**DAI_{BW}**	**MSS****	**DAI_{MSS}**	**Final DAI**
Not noticed	0	0	0	
< 10%	1	<7	1	
10 -15%	2	≥7 but <14	2	$DAI = DAI_{BW} + DAI_{MSS}$
15 - 20%	3	≥14 but <21	3	
> 20%	4	≥21	4	

*BW: Body Weight

**MSS: Murine Sepsis Score

Supplementary Table S2. Antibodies used for flow cytometry

Antigen	Fluorochrome	Concentration	Vendor	Catalog No.
CD45	BV570	1:100	BioLegend/ Thermo Fisher	103136
CD11b	BUV737	1:400	BD	612800
CD3	APC/Cy7	1:20	BD	557596
Ly6G	BV711	1:50	BD	563979
Ly6C	BV421	1:200	BioLegend/ Thermo Fisher	128032
NK1.1	BV786	1:50	eBioscience	417594182
Fixable viability dye	eFluor 506	1:1000	eBioscience	65086614

Supplementary Table S3. Primers used for RT-qPCR analysis of 6-HubG$_{ss}$ gene expression

Primer	Sequence (5'-3')
GAPDH-F	GGAGCGAGATCCCTCCAAAAT
GAPDH-R	GGCTGTTGTCATACTTCTCATGG
GZMB-F	TACCATTGAGTTGTGCGTGGG
GZMB-R	GCCATTGTTTCGTCCATAGGAGA
KLRD1-F	AAAGTCGGCATCTCTGTGCTT
KLRD1-R	CGGTGTGCTCCTCACTGTA
PRF1-F	GACTGCCTGACTGTCGAGG
PRF1-R	TCCCGGTAGGTTTGGTGGAA
SH2D1A-F	AGGCGTGTACTGCCTATGTG
SH2D1A-R	TGCAGAGGTATTACAATGCCTTG
LCK-F	TGCCATTATCCCATAGTCCCA
LCK-R	GAGCCTTCGTAGGTAACCAGT
CD247-F	GGCACAGTTGCCGATTACAGA
CD247-R	CTGCTGAACTTCACTCTCAGG
