# Supplementary material for: Predicting patients with septic shock and sepsis through analyzing whole-blood expression of NK cell-related hub genes using an advanced machine learning framework
Source: Front Immunol. 2024 Nov 28;15:1493895. doi: 10.3389/fimmu.2024.1493895 (PMC11634752; doi:10.3389/fimmu.2024.1493895)
Supplement: Supplementary Data Sheet 2 — The checklist for flow cytometry study. [file DataSheet2.pdf]

frontiers

The International Society for the Advancement of Cytometry (ISAC) has highlighted the importance of including comprehensive methodological information to ensure data reproducibility and reliability. In line with this, Frontiers in Immunology now requires authors to submit a checklist for manuscripts that involve flow or mass cytometry. This checklist helps standardize the reporting process, improving the quality and transparency of published data. By doing so, we support scientific progress, making it easier for other researchers to replicate and validate experiments.

This form should be submitted with any manuscripts using flow or mass cytometry.

Sample/specimen/material description

- [x] Total blood
- [ ] PBMCs
- [ ] Organ digests

Other____

Did the samples suffer any treatment before or after incubation with the antibodies?

- [ ] Drug ______________________________________
- [ ] Cell permeabilization _______________________
- [x] Dye eBioscience™ Fixable Viability Dye eFluor™ 506
- [ ] Propidium iodine
- [ ] Not applicable

Other ____

Instrument and antibodies

Name of the Cytometer BD FACSymphony A5 Cell Analyzer

Antibodies and targets	Fluorochrome/ Metal	Catalog number/Company
e.g. anti-CD4	FITC	Cat. XXX/ XXX ltd.
anti-CD45	BV570	Cat. 103136/BioLegend

anti-CD11b	BUV737	Cat. 612800/BD
anti-CD3	APC/Cy7	Cat. 557596/BD
anti-Ly6G	BV711	Cat. 563979/BD
anti-Ly6C	BV421	Cat. 128032/BioLegend
anti-NK1.1	BV786	Cat. 417594182/

Data analyses

1. Name of the software FlowJo version 10.7.1
2. Reference gating strategy in the manuscript or supplementary material

Gating strategy in (eg Figure X) % of immune cells in CD45+ cells
